# Fistulizing Perianal Disease as a First Manifestation of Crohn’s Disease: A Systematic Review and Meta-Analysis

**DOI:** 10.3390/jcm13164734

**Published:** 2024-08-12

**Authors:** Liesbeth Jozefien Munster, Giulia Louise Emilia Mönnink, Susan van Dieren, Marco William Mundt, Geert Renaat Alfons Maria D’Haens, Willem Adrianus Bemelman, Christianne Johanna Buskens, Jarmila Dagmara Wendelien van der Bilt

**Affiliations:** 1 Department of Surgery, Flevoziekenhuis, 1315 RA Almere, The Netherlands; 2 Department of Surgery, Amsterdam UMC (Location VUMC), 1081 HV Amsterdam, The Netherlandsc.j.buskens@amsterdamumc.nl (C.J.B.); 3 Department of Gastroenterology and Hepatology, Flevoziekenhuis, 1315 RA Almere, The Netherlands; 4 Department of Gastroenterology and Hepatology, Amsterdam UMC (Location VUMC), 1081 HV Amsterdam, The Netherlands

**Keywords:** Crohn’s disease, perianal fistula, delay

## Abstract

**Background**: Incidences of perianal fistulas (PAFs) as a first manifestation of Crohn’s disease (CD) vary widely in the literature. Aim: To analyse the percentage of patients with a PAF preceding CD diagnosis and assess the time to diagnosis. **Methods**: A systematic literature search was conducted. Studies reporting on patients with a PAF preceding CD diagnosis were identified. Primary outcomes were the (weighted) percentage of patients with CD with a PAF preceding CD and their time to CD diagnosis. Secondary outcomes were the (weighted) percentage of patients with CD with a PAF preceding CD diagnosis in predefined patient subgroups, including (1) sex (men vs. women), (2) ethnicity (Asian vs. non-Asian), and (3) age (paediatric (0–18 y) and patients with elderly onset CD (>60 y) vs. adult patients (18–60 y)). **Results**: Seventeen studies were included (34,030 patients with CD). In the overall CD population, a PAF preceded CD in 8.6% [95%CI; 5.72; 12.71] with a weighted mean time to CD diagnosis of 45.9 (31.3) months. No studies reported details on sex differences in patients with a PAF as a manifesting sign of CD. In Asian populations, a PAF preceded CD in 17.66% [95%CI; 11.45; 26.25], which was significantly higher when compared with non-Asians (4.99% [95%CI; 3.75; 6.60], OR:3.99, *p* < 0.0001). In adolescents, an incidence of 9.17% [95%CI; 5.92; 13.93] was found with significantly lower incidences in paediatric patients (6.38% [95%CI; 1.84; 19.85], OR:0.53, *p* < 0.0001), and elderly-onset patients (3.77% [95%CI; 1.68; 8.25], OR:0.44, *p* = 0.0035). **Conclusions**: This systematic review shows that in the literature, almost 10% of patients present with a PAF as a first manifestation of CD, with a mean time to diagnosis of almost four years. These results emphasise that increased clinical awareness is needed.

## 1. Introduction

Perianal fistulas (PAFs) are common in Crohn’s disease (CD) and are regarded as one of the most debilitating symptoms of the disease [1,2,3,4]. Approximately one-third of all patients with CD will be confronted with a PAF during the disease course, either before or after their CD diagnosis [1,5]. Reported incidences of CD related PAFs and timing of manifestations seem to vary widely within studies and various subgroups of patients (disease phenotype, patient’s country of origin, and age) [6,7]. Perianal symptoms may precede intestinal symptoms in CD for many years and may occur without any luminal signs [8,9,10,11,12,13]. Interestingly, time to CD diagnosis in patients with a PAF as a first manifestation is reported to be much longer than in patients presenting with luminal complaints [14,15]. Moreover, the literature states that the presence of PAFs at CD diagnosis is associated with a poor prognosis and an elevated risk of a disabling and complex disease course [16,17]. It was shown that combining anti-tumour necrosis factor (anti-TNF) therapy with surgical closure offers the highest chance of radiological healing, which nowadays is assumed to be the highest therapeutic goal for all PAF patients [18,19,20,21,22]. Correspondingly, a long diagnostic delay will result in delayed initiation of appropriate therapy, probably impacting outcomes [18], not only for the perianal disease but also with increased risk of bowel stenosis and need for surgical intervention in case of luminal disease [16].

Studies specifically focusing on patients with PAF as a manifesting sign of CD are limited, and it is difficult to draw firm conclusions on the true incidence of PAF as a first manifestation. Moreover, the relevance for daily clinical practice and its impact on long-term outcomes remains unknown. This current study aimed to analyse the percentage of patients with a PAF preceding CD diagnosis and assess their time to diagnosis by reviewing the current literature. In addition, the impact of time to diagnosis on long-term clinical outcomes was analysed.

## 2. Materials and Methods

### 2.1. Search Strategy

A systematic literature search was conducted for articles reporting on patients with a PAF preceding CD diagnosis according to PRISMA (Preferred Reporting Items for Systematic Reviews and Meta-analyses) guidelines [23]. A detailed protocol was registered in the PROSPERO database (ID: CRD42022365616). Guided by an experienced medical librarian, the search was conducted in the following databases: Pubmed, EMBASE, Cochrane, and Web of Science. The search strategy included (MeSH) terms and free text related to or describing ‘Crohn’s disease’, ‘perianal diseases’, ‘diagnosis’, and/or ‘incidence’ or ‘prevalence’. No study date restrictions were applied. Animal studies, case reports, reviews, letters, conference abstracts, editorials, and comments were excluded. The final search was conducted on 28 August 2023. A detailed report of the search strategy is provided in Supplementary Table S1.

### 2.2. In-And Exclusion Criteria

Studies meeting the following criteria were included: (1) concerning a general CD cohort as the study population, (2) describing patients with a PAF preceding CD diagnosis [24] (i.e., PAF first), and (3) including >10 patients with CD. 

In case different articles used the same study population in their analysis (e.g., same database and the same period of time), only one was included to prevent overrepresentation of the study population in the analysis. 

Studies were excluded when they (1) did not distinguish between the type of perianal lesion (e.g., skin tag, PAF, PAA, fissure, or haemorrhoid), (2) included rectovaginal fistulas, (3) reported on the incidence of CD-related PAFs in a general PAF cohort solely, or (4) were in languages other than English or Dutch (due to logistic reasons).

## 3. Outcomes

Primary outcomes were the percentage of patients with a PAF preceding CD diagnosis (presented as a weighted mean percentage) and their associated time to diagnosis. Secondary outcomes were the (weighted) percentage of patients with CD with a PAF preceding CD diagnosis in predefined patient subgroups, including (1) sex (men vs. women), (2) ethnicity (Asian vs. non-Asian), and (3) age (paediatric (0–18 y) and patients with elderly onset CD (>60 y) vs. adult patients (18–60 y)). Additionally, time to diagnosis was correlated to length of follow-up (to correct for possible confounding) and long-term clinical outcomes. The quality of all studies was assessed by the use of the Newcastle Ottawa scale (NOS) [25].

### 3.1. Study Selection and Data Extraction

Two reviewers (GM and LM) screened titles and abstracts independently by the use of Rayyan [26]. Any disagreements in the selection process were resolved by discussion, and if necessary, a third researcher (JB) was consulted. After title and abstract screening, full texts were screened, and articles were evaluated in-depth to include studies specifically describing patients with a PAF preceding CD diagnosis and, if mentioned, their time to diagnosis (including the correlation between time to diagnosis and long-term clinical outcomes if reported). 

Study characteristics (author, year of publication, country of publication, study design, study span, data source, study cohort, percentage of patients with CD used in the analysis, CD cohort subtypes) and patient characteristics (age and gender) were extracted. All data were recorded in a Microsoft Excel database (Microsoft Office version 2016). All missing data were reported and were handled by only analysing available data in the literature. 

### 3.2. Statistical Analysis 

Categorical data were presented as counts and percentages. Continuous data were presented as median and interquartile range (IQR) or means and standard deviation (SD). Time to diagnosis reported as median was converted to approximated mean values using a method suggested by Wan et al. [27] To standardise time units used in different studies, all data were converted to months before analysis. Meta-analyses were performed to calculate a weighted mean percentage [CI 95%] using the inverse variance method and a random effect model for the primary and secondary outcomes. Heterogeneity among the included studies was evaluated by computing the inconsistency index (I^2^). Values >50% indicated significant heterogeneity [28]. A Pearson’s correlation coefficient (r) was determined to assess the relationship between the number of patients with PAF first and the total number of patients with CD. Univariate analyses were used to determine the odds ratios (ORs) and 95% confidence intervals (CIs) of the variables associated with a PAF preceding CD. In case different studies were compared, a meta-regression on the random effects was performed. A two-tailed *p*-value of less than 0.05 was considered significant. All analyses were performed using Statistical Package for the Social Sciences (SPSS) for Windows (version 28, IBM Crop., Armonk, NY, USA) and RStudio for Windows (version 4.2.1). 

## 4. Results 

### 4.1. Study Selection 

**Figure 1:**
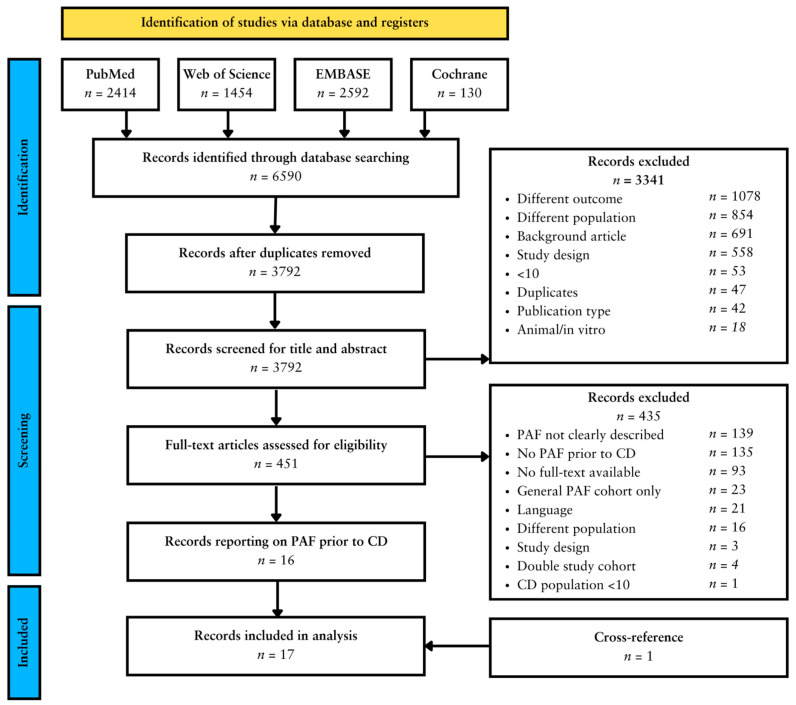
Study selection process according to PRISMA guidelines [23].

In the final search, a total of 6590 articles were retrieved. After the removal of duplicates, 3792 abstracts were included for screening. A total of 451 articles remained eligible for full-text screening. Among these, sixteen articles reported specifically on unique cohorts of patients with a PAF preceding CD diagnosis [29,30,31,32,33,34,35,36,37,38,39,40,41,42,43,44]. Cross-referencing yielded one additional article [45], which was included in the final analysis, resulting in a total of seventeen articles to be included. A comprehensive visual representation of the study selection process is presented in Figure 1. 

### 4.2. Study Characteristics and Quality Assessment 

Comprehensive study details of all seventeen studies included are summarised in Table 1. All studies (*n* = 17) were of a retrospective nature, of which nine were conducted in a multicenter setting. The studies included a total of 34,030 patients with CD (35% male), with cohort sizes ranging from 63 patients [32] to 12,905 patients [29]. The majority of studies report on adult patients. Two studies primarily focused on elderly onset CD [31,40], while two other studies exclusively concerned paediatric patients with CD [32,35]. Overall quality of the studies was moderate to good. Especially the length of follow-up and the adequacy of follow-up were lacking or insufficiently reported in most studies. For an overview of the results of the critical appraisal, see Supplementary Table S2. 

### 4.3. PAF First in Patients with CD and Time to Diagnosis of CD

**Figure 2:**
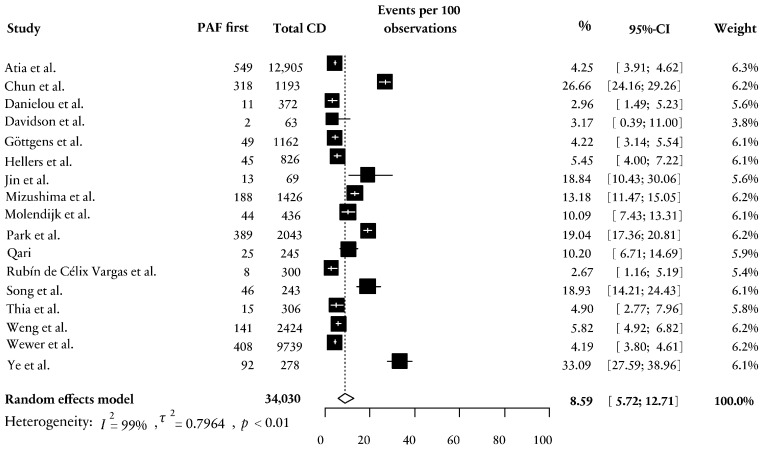
Forest plot on the weighted mean percentage of PAF first in patients with CD [29,30,31,32,33,34,35,36,37,38,39,40,41,42,43,44,45]. PAF = perianal fistula; CD = Crohn’s disease; CI = confidence interval; *p* = significance.

Seventeen studies reported on patients with a PAF as the first manifestation within the general CD cohort (Table 1) [29,30,31,32,33,34,35,36,37,38,39,40,41,42,43,45,46]. The study population comprised 34,030 patients with CD, with 2343 experiencing a PAF prior to their CD diagnosis. The incidence of a PAF before CD diagnosis ranged from 3 to 33% with a weighted mean of 8.59% [95% CI; 5.72; 12.71], r = 0.838, *n* = 17, *p* =< 0.001, Figure 2). Among the included studies, seven provided information on the time to diagnosis of CD after PAF manifestation. Five of these studies presented the time to diagnosis as either median (IQR/range) and/or mean (SD) values and were included in the analysis [33,36,41,42,44]. Four studies defined preceding diagnosis as the development or diagnosis of a PAF prior to CD diagnosis, and one study specifically described it as the development of a PAF > 6 months prior to CD diagnosis [42]. Time to diagnosis of CD ranged from 10.8 (15.8) months [36] to 106.75 (72.7) months [44], with a weighted mean time to diagnosis of 45.9 (31.3) months. In Supplementary Table S3, an overview of the (converted) times to diagnosis is provided.

### 4.4. PAF First and Sex

Although four studies reported on male predominance for PAF in general [40,42,44,45], no studies reported details on sex differences specifically in patients with a PAF as a manifesting sign of CD.

### 4.5. PAF First and Ethnicity

**Figure 3:**
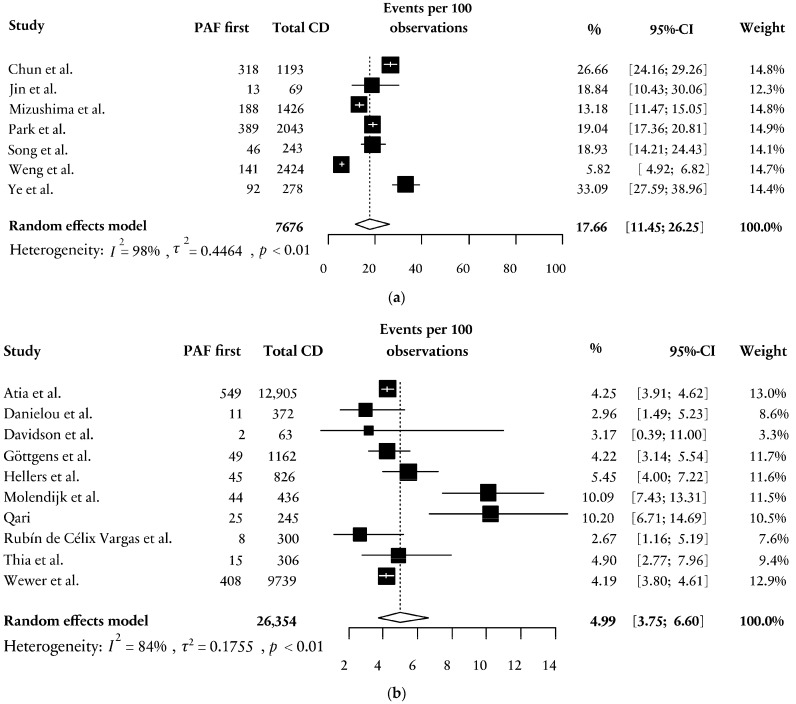
(**a**) Forest plot on the weighted percentage of PAF first in patients with CD in Asian cohort studies [30,35,36,40,42,44,45]. PAF = perianal fistula; CD = Crohn’s disease; CI = confidence interval; *p* = significance. (**b**) Forest plot on the weighted percentage of PAF first in patients with CD in non-Asian cohort studies (reference group) [29,31,32,33,34,37,38,39,41,43]. PAF = perianal fistula; CD = Crohn’s disease; CI = confidence interval; *p* = significance.

A subanalysis was performed to compare the proportion of PAF-first patients in Asian populations as compared to non-Asian populations. Seven Asian studies identified a total of 1187 patients with a PAF first among 7676 patients with CD, resulting in a weighted mean of 17.66% [95% CI; 11.45; 26.25], Figure 3a [30,35,36,40,42,44,45]. This was significantly higher (OR 3.99 [95% CI; 3.66; 4.34], *p* < 0.0001) than the 4.99% [95% CI; 3.75; 6.60] found in the analysis of ten non-Asian studies, Figure 3b [29,31,32,33,34,37,38,39,41,43].

### 4.6. PAF First and Age

**Figure 4:**
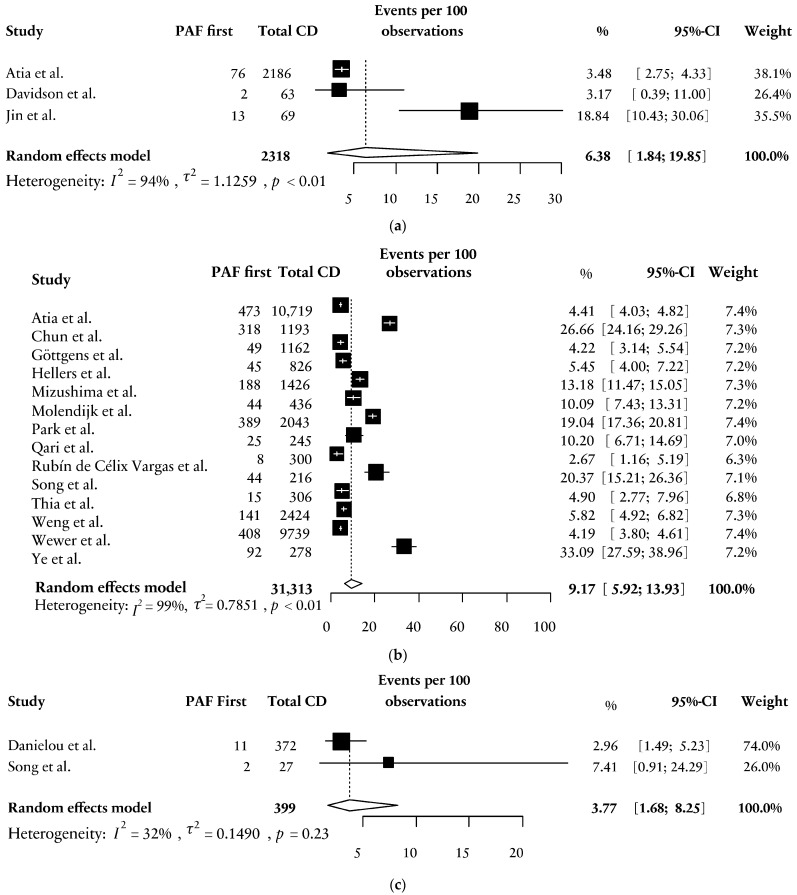
(**a**) Forest plot on the weighted percentage of PAF first in paediatric patients [29,32,35]. PAF = perianal fistula; CD = Crohn’s disease; CI = confidence interval; *p* = significance. (**b**) Forest plot on the weighted percentage of PAF first in patients 18–60 years (reference group) [29,30,33,34,36,37,38,39,40,41,42,43,44,45]. PAF = perianal fistula; CD = Crohn’s disease; CI = confidence interval; *p* = significance. (**c**) Forest plot on the weighted percentage of PAF first in patients with elderly-onset CD (>60 years) [31,40]. PAF = perianal fistula; CD = Crohn’s disease; CI = confidence interval; *p* = significance.

Three studies, either focusing on paediatric patients or concerning paediatric subcohorts, were included [29,32,35]. When assessing the proportion of children with a PAF first as a fraction of all paediatric patients with CD (*n* = 2.318), a weighted mean of 6.38% [95% CI; 1.84; 19.85] was found, which was significantly lower when compared with 9.17% [95% CI; 5.92; 13.93] in patients from 18–60 years who served as reference (OR 0.53 [95% CI; 0.43; 0.66], *p* < 0.0001). In addition, two studies that addressed a PAF first in patients with elderly-onset CD (>60 years) revealed a weighted mean of 3.77% [95% CI;1.68; 8.25] [31,40], which was also significantly lower than the results as shown in the reference group (OR 0.44 [95% CI 0.25; 0.76], *p* = 0.0035, Figure 4).

### 4.7. Time to Diagnosis Correlated to Length of Follow-Up

Supplementary Figure S1 shows the percentage of patients with a PAF prior to CD diagnosis plotted against mean follow-up in years. It was shown that the eventual percentage of patients with a PAF prior to CD diagnosis was not correlated to the length of follow-up of included studies.

### 4.8. Impact of Time to Diagnosis on Long-Term Clinical Outcomes

Six studies reported on clinical outcomes in patients with perianal CD [33,36,39,42,43,45]. One study showed that patients with perianal CD had a significantly increased likelihood of undergoing (major) abdominal surgeries (including ostomy and proctectomy/proctocolectomy) [43]. Two other studies identified PAF prior to CD diagnosis as a poor prognostic factor [36,45] and suggested that early CD diagnosis could improve outcomes. However, no details were provided, and no correlation between time to diagnosis and clinical outcomes was reported. The remaining three studies presented fistula recurrences but did not discriminate fistula-first patients from the overall CD fistula population [33,39,42].

## 5. Discussion

This systematic review shows that a PAF as a first manifestation of CD occurs in 8.6% of all patients with CD, equaling around one-third of all patients with CD-related PAF. The weighted mean time to CD diagnosis in these patients was long, with an estimation of 45.9 (31.3) months. The percentage of patients with a PAF as a first manifestation was higher in the Asian population as compared with the non-Asian population (18% versus 5%, respectively), and adult patients with CD as compared with paediatric patients with CD and patients with elderly-onset CD (9% versus 6% and 4%, respectively).

The percentage of patients with a PAF as a manifesting sign of CD in this study is considerably higher than those reported in a recent systematic review by Tsai et al. [46], which investigated the cumulative incidence of perianal disease. In that study, it was reported that 3.8% (based on five studies, 95% CI 1.9–7.3%) of patients with CD developed perianal disease prior to luminal CD diagnosis. A possible explanation for this difference is that the current study included seventeen studies reporting on patients with a PAF first within the general CD cohort, which may have led to a more reliable percentage.

Time to CD diagnosis in patients with a PAF as a first manifestation is reported to be much longer than in patients presenting with luminal complaints [14,15]. Although none of the included studies provided details on the correlation between time to diagnosis and long-term clinical outcomes, several reports suggested that longer delays were associated with worse clinical outcomes [14,47], which underscores the need for action and the development of screening tools for early identification of patients at high risk of having CD. In addition, the start of adequate therapy will also be beneficial on the QoL of these patients, as fistula impact is known to be substantial, with symptoms such as faecal incontinence, rectal pain, and swelling affecting patients’ daily lives and sexual activity [48,49].

Recently, the International Organization for the Study of Inflammatory Bowel Disease (IO-IBD) identified ‘Red Flags’ suggestive for patients with CD, showing that a non-healing or complex PAF was strongly associated with the diagnosis of CD (OR 50.7) (95% CI 6.7–382.7) (*p* < 0.0001) [50]. Unfortunately, it remains challenging in clinical practice to be attentive to underlying CD in patients who present with a PAF as the sole manifestation. Despite that new insights in treatment for CD-related PAF combining anti-TNF therapy and surgical closure have improved prognosis, overall outcomes remain suboptimal, impacting both QoL and healthcare costs [19,20,21,22]. Awareness of this problem needs to be raised, as it not only leads to diagnostic delays but also mainly affects young adults who are in the midst of their socioeconomic lives [33,36]. The findings in this study underscore that a substantial part of patients with CD have PAF prior to CD diagnosis.

An interesting finding was that patients originating from Asian countries showed higher percentages of patients presenting with a PAF as the manifesting symptom of CD as compared with patients originating from non-Asian countries. A recent systematic review investigating the influence of ethnicity on phenotype and outcome in IBD showed that Asian and African patients with CD had more perianal involvement compared with Caucasian and/or Hispanic patients [6]. In addition, a large study on the disease phenotype of Korean paediatric patients with CD found that they had a higher probability of experiencing PAF at diagnosis than their peers from Europe (44.8% vs. 8.2%, *p* < 0.001) [7]. A potential explanation is that *TNFSF15* polymorphisms (a so-called IBD gene that is upregulated in lymphocytes as well as in macrophages of the intestinal lamina propria in patients with CD), which are common in Asian populations, are independent predictive risk factors for the development of PAF, and are therefore associated with a higher risk of PAF [51,52]. Nevertheless, more research on the influence of ethnicity on disease phenotype needs to be conducted.

This study also demonstrated that paediatric patients presented less often with a PAF as a first manifestation of CD, which conforms to the literature stating that the risk of having CD increases with age when presenting with perianal disease [53,54,55,56,57]. In line with this, Roskam et al. [54] showed that the risk of CD in infants presenting with perianal disease solely is low. Moreover, it was shown that paediatric patients often present with isolated (ileo-)colonic disease without perianal involvement, which may have led to the lower percentages of patients with a PAF as a first manifestation [55]. Also, this study showed that PAF as a manifesting sign was frequently less seen in patients with elderly onset CD, which also conforms to the literature showing that patients with elderly onset CD were more likely to present with a colonic disease phenotype without involvement of the perianal region [56].

One of the major strengths of this study was that this study included a general population of patients with CD, which is most likely to be encountered in daily clinical practice. While, to our knowledge, this systematic review is the first to investigate the percentage of patients with CD with a PAF prior to CD diagnosis and their time to diagnosis, there are several limitations inherent to its design. Heterogeneity between studies was high with respect to cohort size, follow-up time, and quality. To avoid heterogeneity due the various definitions of perianal CD (which might also include fissures, skin tags, etc.), it was decided to only include studies presenting results of patients with PAF prior to CD diagnosis and no other perianal diseases. Unfortunately, the specific timeframe for ‘prior to diagnosis’ was not always specified, which may have contributed to the high heterogeneity rates in this study. However, it was shown that cohort sizes, as well as the length of follow-up in all studies, had no effect on the correlation between patients with a PAF first and the total CD cohort in all (sub)analyses. Still, as with all systematic reviews, it is important to interpret results with caution as all included studies were of retrospective design and its inherent limitations. Additionally, the quality assessment was of limited value due to the inapplicability of some of the questions in the NOS critical appraisal. In the current study, it was shown that the eventual percentage of patients with a PAF prior to CD diagnosis was not correlated to the length of follow-up, which is remarkable since literature states that a longer disease period is associated with an increased probability of developing PAF [57]. Since the majority of all included studies did not elaborate on follow-up periods, it may be possible that the percentage of patients with CD presenting with a PAF as a first manifestation is an underestimation of the real number of patients.

In conclusion, this systematic review shows that in the literature, almost 10% of patients present with a PAF as a first manifestation of CD with a substantial delay in diagnosis. These results emphasise that increased clinical awareness is warranted in order to decrease delay in CD diagnosis.

## Figures and Tables

**Table 1:** Overview of the included studies.

Author	Year	Country	Study Design	Single or Multicenter	Study Span (y)	Data Source	CD Patients (*n*)	Subtypes CD Cohort	Subtypes CD Cohort (*n*, %)	Age (y)	Male (*n*, %)	PAF Prior to CD Diagnosis (*n*, %)	Diagnostic Delay (Median)	Follow-Up (Median)
Atia et al. [29]	2022	Israel	Retrospective population-based cohort study	Multicenter	2005–2019	epi-IIRN	12,905	(1) Adults; (2) children	(1) 10,719 (83) (2) 2186 (17)	(1) 34.4 (13.8) *; (2) 14.1 (3.5) *	(1) 6646 (62); (2) 1661 (76)	(1) 473/10,719 (4.4); (2) 76/2186 (3.5)	NR	7.8 y [IQR 4.2–11.0]
Chun et al. [30]	2018	Korea	Retrospective cohort study	Multicenter	1982–2008	CONNECT study database	1193	NA	NA	26.9 (11.9) *	840 (70.4)	318/1193 (26.7)	NR	8.77 y (1.0 to 25.8) **
Danielou et al. [31]	2020	France	Retrospective population-based cohort study	Multicenter	1988–2006	EPIMAD registry	372	NA	NA	70.1 [65.2–76.4]	142 (38.2)	11/372 (3)	NR	6 y [IQR 3–10]
Davidson et al. [32]	1992	Australia	Retrospective cohort study	Single center	1971–1987	Medical records	63	NA	NA	12 [3–16]	11 (17.5)	2/63 (3.2)	NR	NR
Göttgens et al. [33]	2016	The Netherlands	Retrospective population-based cohort study	Multicenter	1991–2011	IBDSL registry	1162	(1) Without PAF/RVF; (2) only PAF; (3) RVF	(1) 995 (85.6); (2) 150 (12.9); (3) 17 (1.5)	(1) 38.5 (16.3) *; (2) 32.3 (12.5) *; (3) 37.3 (15.1) *	(1) 375 (37.7); (2) 59 (39.3); (3) 0 (0)	49/1162 (4.2)	0.8 y [0.2–2.7]	8.7 y (5.7) **
Hellers et al. [34]	1980	Sweden	Retrospective cohort study	Multicenter	1955–1974	Medical records	826	NA	NA	NR	379 (45.9)	45/826 (5.4)	(1) >2 y prior to CD diagnosis (19); (2) 6 m > and >2 y (26)	9.4 y (0.5–22.5) **
Jin et al. [35]	2018	Korea	Retrospective cohort study	Single center	2000–2014	Medical records	69	(1) CD with perianal lesions; (2) CD without perianal lesions	(1) 54 (78.2); (2) 15 (21.7)	15.4	51 (73.9)	13/69 (18.8)	14 m	NR
Mizushima et al. [36]	2021	Japan	Retrospective cohort study	Multicenter	2013–2019	JMDC Co., Ltd., claims database	1426	(1) PAF after CD; (2) CD after PAF; (3) PAF + CD ^†^; (4) CD only	(1) 43 (3.0); (2) 188 (13.2); (3) 43 (3.0); (4) 1152 (80.8)	(1) 27.1 (12.7) *; (2) 25.7 (10.5) *; (3) 26.9 (13.6) *; (4) 35.9 (15.7) *	(1) 39 (90.7); (2) 169 (89.9); (3) 36 (83.7) (4) 757 (65.7)	188/1426 (13.2)	10.8 m (15.8) **	≥12 m
Molendijk et al. [37]	2014	The Netherlands	Retrospective cohort study	Single center	1980–2000	Medical records	436	NA	NA	22.8 [4.0–68.7] *	NR	44/436 (10.1) *	NR	NR
Park et al. [45]	2014	Korea	Retrospective cohort study	Single center	1981–2012	Medical records	2043	(1) 1981–2000; (2) 2001–2005; (3) 2006–2012	(1) 363 (17.8); (2) 611 (29.9); (3) 1069 (52.3)	23 [9–75] *	1462 (71.6)	(1) 70/363 (19.3); (2) 118/611 (19.3); (3) 201/1069 (18.8)	NR	80 m (1–381)
Qari [38]	2022	Saudi Arabia	Retrospective cohort study	Single center	2012–2018	Medical records	245	NA	NA	26.3 [14–73] *	125 (51)	25/245 (10.2)	NR	NR
Rubín de Célix Vargas et al. [39]	2018	Spain	Retrospective cohort study	Single center	2004–2016	Medical records	300	NA	NA	NR	36 (12)	8/300 (2.7)	NR	NR
Song et al. [40]	2018	Korea	Retrospective matched case-control study	Single center	1989–2016	Asan IBD registry	243	(1) Elderly onset; (2) Middle-age onset; (3) Young onset	(1) 27 (11.1); (2) 108 (44.4); (3) 108 (44.4)	26.0 [21.0–34.1] *	161 (66.3)	(1) 2/27 (7.4); (2) 15/108 (13.9); (3) 29/108 (26.9)	NR	67.8 m [IQR 40.5–120.8]
Thia et al. [41]	2010	USA	Retrospective cohort study	Multicenter	1970–2004	Medical records (Olmsted County Database)	306	NA	NA	30.2 [3–142]*	150 (49)	15/306 (4.9)	38 m [range, 3–142]	8.4 y [2 d–35.9 y]
Weng et al. [42]	2023	Taiwan	Retrospective cohort study	Multicenter	2000–2017	Taiwan’s National Health Insurance Research Database	2424	(1) With pCDl; (2) without pCD	(1) 358 (14.8); (2) 2066 (85.2)	(1) 33.7 (14.9); (2) 44.9 (21.8)	(1) 284 (79.3); (2) 1248 (60.4)	141/2424 (5.8)	(1) 1266 [756–2237]; (2) 1611 d (1213) **	Not clearly reported
Wewer et al. [43]	2021	Denmark	Retrospective cohort study	Multicenter	1997–2015	National Patient Registry	9739	(1) Without pCD; (2) with pCD	(1) 7927 (81.4); (2) 1812 (18.6)	(1) 39.6 [27.0–57.0] *; (2) 32.8 [24.1–46.2] *	(1) 3317 (41.8); (2) 903 (49.8)	408/9739 (4.2)	NR	8.2 y [4.1–13.3]
Ye et al. [44]	2010	Korea	Retrospective cohort study	Single center	1991–2007	Medical records	278	NA	NA	23 [9–74] *	191 (68.7)	92/278 (33.1)	32 [2–361] m	71 m [1–210]

Data provided as *n* (%). * = age at CD diagnosis; ** = mean (SD); IBD = Inflammatory Bowel Disease; CD = Crohn’s disease; PAF = perianal fistula; pCD = perianal Crohn’s disease; NA = not applicable, NR = not reported; y = year; m = month; ^†^ = same month.

## Data Availability

The authors of this manuscript confirm that the data supporting the findings of this systematic review are available within this manuscript or in its Supplementary Materials. Additional details can be provided upon request.

## Supplementary Materials

The following supporting information can be downloaded at: https://www.mdpi.com/article/10.3390/jcm13164734/s1, Figure S1: Proportion of patients with a PAF prior to CD diagnosis plotted against mean follow-up; Table S1: Search strategy; Table S2: Overview of the critical appraisal results. NA = not applicable; Table S3: Time to diagnosis of CD after PAF manifestation as reported in studies and converted to a mean time to diagnosis in months using the method by Wan et al. [27]. * = median delay as was reported in the study; y = years; m = months; d = days; NR = not reported; sample size = number of patients in whom time to CD diagnosis was reported.
