# Supplementary material for: Fistulizing Perianal Disease as a First Manifestation of Crohn’s Disease: A Systematic Review and Meta-Analysis
Source: J Clin Med. 2024 Aug 12;13(16):4734. doi: 10.3390/jcm13164734 (PMC11355404; doi:10.3390/jcm13164734)
Supplement: Supplementary file 1 [file jcm-13-04734-s001.zip › Supplementary Table S1 - Search strategy.pdf]

PubMed (2,414)

Search	Query	Results
#7	Search: **#6 NOT ("Animals"[Mesh] NOT "Humans"[Mesh])**	2,414
#6	Search: **#5 NOT ("Case Reports"[Publication Type] OR "Letter"[Publication Type] OR "Editorial"[Publication Type] OR "Comment"[Publication Type])**	2,421
#5	Search: **#1 AND #2 AND (#3 OR #4)**	2,950
#4	Search: **"Incidence"[Mesh] OR "Prevalence"[Mesh] OR prevalence*[tiab] OR Incidence*[tiab] OR "New case*"[tiab]**	1,860,938
#3	Search: **"Diagnosis"[Mesh] OR "diagnosis"[Subheading] OR Diagnos*[tiab] OR "first manifestation*"[tiab] OR "Manifesting sign*"[tiab] OR "At onset"[tiab]**	11,722,526
#2	Search: **"Rectal Fistula"[Mesh] OR Perianal*[tiab] OR "Perianal dis*"[tiab] OR "Perianal abscess*"[tiab] OR "Perianal fistul*"[tiab] OR "Perianal crohn's"[tiab] OR "Perianal crohn s"[tiab] OR "Perianal crohns"[tiab] OR "Perianal lesion*"[tiab] OR "Perianal symptom*"[tiab] OR "Perianal complication*"[tiab] OR "External fistul*"[tiab] OR "Anoperineal lesion*"[tiab] OR "perineal*"[tiab] OR "Rectal dis*"[tiab] OR "Rectal abscess*"[tiab] OR "Rectal fistula*"[tiab] OR "Anal dis*"[tiab] OR "Anal abscess*"[tiab] OR "Anal fistula*"[tiab] OR "Anal complication*"[tiab] OR "Anal lesion*"[tiab] OR "External fistulae"[tiab] OR "Fistula formation*"[tiab] OR "Cryptoglandular fistula*"[tiab]**	36,728
#1	Search: **"Inflammatory Bowel Diseases"[Mesh] OR "Crohn Disease"[Mesh] OR "Inflammatory Bowel Dis*"[tiab] OR IBD[tiab] OR Crohn*[tiab] OR "Regional Enteriti*"[tiab] OR "Granulomatous Enteriti*"[tiab] OR "Ileocolitis"[tiab] OR "Granulomatous Colitis"[tiab] OR "Terminal Ileitis"[tiab] OR "Regional Ileiti*"[tiab]**	135,615

Embase (2,592)

Search	Query	Results
#6	Search: **#5 NOT ("Case Reports"[Publication Type] OR "Letter"[Publication Type] OR "Editorial"[Publication Type] OR "Comment"[Publication Type])**	2,592
#5	**#1 AND #2 AND (#3 OR #4)**	5,881
#4	'prevalence'/exp OR 'incidence'/exp **OR (prevalence* OR Incidence* OR "New case*"):ti,ab,kw**	2,803,181
#3	'diagnosis'/exp **OR (Diagnos* OR "first manifestation*" OR "Manifesting sign*" OR "At onset"):ti,ab,kw**	10,275,199
#2	**'rectum fistula'/exp OR (Perianal* OR "Perianal dis*" OR "Perianal abscess*" OR "Perianal fistul*" OR "Perianal crohn s" OR "Perianal crohn s" OR "Perianal crohns" OR "Perianal lesion*" OR "Perianal symptom*" OR "Perianal complication*" OR "External fistul*" OR "Anoperineal lesion*" OR "perineal*" OR "Rectal dis*" OR "Rectal abscess*" OR "Rectal fistula*" OR "Anal dis*" OR "Anal abscess*" OR "Anal fistula*" OR "Anal complication*" OR "Anal lesion*" OR "External fistulae" OR "Fistula formation*" OR "Cryptoglandular fistula*"):ti,ab,kw**	54,818
#1	'inflammatory bowel disease'/exp **OR 'Crohn disease'/exp OR ("Inflammatory Bowel Dis*" OR IBD OR Crohn* OR "Regional Enteriti*" OR "Granulomatous Enteriti*" OR "Ileocolitis" OR "Granulomatous Colitis" OR "Terminal Ileitis" OR "Regional Ileiti*"):ti,ab,kw**	245,774

Web of Science (1,454)

Search	Query	Results
#6	**#1 AND #2 AND (#3 OR #4)** and **Proceeding Paper** or **Meeting Abstract** or **Early Access** or **Editorial Material** or **Letter** or **Book Chapters** (Exclude – Document Types)	1,454
#5	**#1 AND #2 AND (#3 OR #4)**	1,603
#4	TS=(**prevalence* OR Incidence* OR "New case*"**)	2,054,587
#3	TS=(**Diagnos* OR "first manifestation*" OR "Manifesting sign*" OR "At onset"**)	3,244,519

#2	TS=(Perianal* OR "Perianal dis*" OR "Perianal abscess*" OR "Perianal fistul*" OR "Perianal crohn's" OR "Perianal crohn s" OR "Perianal crohns" OR "Perianal lesion*" OR "Perianal symptom*" OR "Perianal complication*" OR "External fistul*" OR "Anoperineal lesion*" OR "perineal*" OR "Rectal dis*" OR "Rectal abscess*" OR "Rectal fistula*" OR "Anal dis*" OR "Anal abscess*" OR "Anal fistula*" OR "Anal complication*" OR "Anal lesion*" OR "External fistulae" OR "Fistula formation*" OR "Cryptoglandular fistula*")	32,309
#1	TS=("Inflammatory Bowel Dis*" OR IBD OR Crohn* OR "Regional Enteriti*" OR "Granulomatous Enteriti*" OR "Ileocolitis" OR "Granulomatous Colitis" OR "Terminal Ileitis" OR "Regional Ileiti*")	161,496

Cochrane (130)

Search	Query	Results
#5	#1 AND #2 AND (#3 OR #4)	130
#4	(prevalence* OR Incidence* OR New NEXT case*):ti,ab,kw	186,249
#3	(Diagnos* OR first NEXT manifestation* OR Manifesting NEXT sign* OR At NEXT onset):ti,ab,kw	299,448
#2	(Perianal* OR Perianal NEXT dis* OR Perianal NEXT abscess* OR Perianal NEXT fistul* OR Perianal NEXT crohn's OR Perianal NEXT crohn NEXT s OR Perianal NEXT crohns OR Perianal NEXT lesion* OR Perianal NEXT symptom* OR Perianal NEXT complication* OR External NEXT fistul* OR Anoperineal NEXT lesion* OR perineal* OR Rectal NEXT dis* OR Rectal NEXT abscess* OR Rectal NEXT fistula* OR Anal NEXT dis* OR Anal NEXT abscess* OR Anal NEXT fistula* OR Anal NEXT complication* OR Anal NEXT lesion* OR External NEXT fistulae OR Fistula NEXT formation* OR Cryptoglandular NEXT fistula*):ti,ab,kw	4,496
#1	(Inflammatory NEXT Bowel NEXT Dis* OR IBD OR Crohn* OR Regional NEXT Enteriti* OR Granulomatous NEXT Enteriti* OR Ileocolitis OR Granulomatous NEXT Colitis OR Terminal NEXT Ileitis OR Regional NEXT Ileiti*):ti,ab,kw	8,708
#5	#1 AND #2 AND (#3 OR #4)	130
#4	(prevalence* OR Incidence* OR New NEXT case*):ti,ab,kw	186,249

Supplementary Table S1. Search strategy.
