# Supplementary material for: Fistulizing Perianal Disease as a First Manifestation of Crohn’s Disease: A Systematic Review and Meta-Analysis
Source: J Clin Med. 2024 Aug 12;13(16):4734. doi: 10.3390/jcm13164734 (PMC11355404; doi:10.3390/jcm13164734)
Supplement: Supplementary file 1 [file jcm-13-04734-s001.zip › Supplementary Table S2 - Overview of the critical appraisal results.pdf]

	Selection				Comparability	Outcome			
NOS Cohort studies on CD cohorts	Representativeness of exposed cohort	Selection of non-exposed cohort	Ascertainment of exposure	Demonstration that outcome of interest was not present at start of study	Comparability of cohorts based on design or analysis	Assessment of outcome	Was follow-up long enough for outcomes to occur	Adequacy of follow up of cohorts	Total (5/5)
Atia et al. [29]	☆	NA	☆	NA	NA	☆	☆	0	4/5
Chun et al. [30]	☆	NA	☆	NA	NA	☆	☆	☆	5/5
Danielou et al. [31]	☆	NA	☆	NA	NA	☆	☆	0	4/5
Davidson et al. [32]	☆	NA	☆	NA	NA	☆	0	0	3/5
Göttgens et al. [33]	☆	NA	☆	NA	NA	☆	☆	0	4/5
Hellers et al. [34]	☆	NA	☆	NA	NA	☆	☆	☆	5/5
Jin et al. [35]	☆	NA	☆	NA	NA	☆	0	0	3/5
Mizushima et al. [36]	☆	NA	☆	NA	NA	☆	0	0	3/5
Park et al. [45]	☆	NA	☆	NA	NA	☆	☆	☆	5/5
Qari [38]	☆	NA	☆	NA	NA	☆	0	0	3/5
Rubín de Célix Vargas et al. [39]	☆	NA	☆	NA	NA	☆	0	0	3/5
Thia et al. [41]	☆	NA	☆	NA	NA	☆	☆	0	4/5
Weng et al. [42]	☆	NA	☆	NA	NA	☆	☆	0	4/5
Wewer et al. [43]	☆	NA	☆	NA	NA	☆	☆	☆	5/5
Ye et al. [44]	☆	NA	☆	NA	NA	☆	☆	0	4/5
Molendijk et al. [37]	☆	NA	☆	NA	NA	☆	☆	0	4/5

	Selection				Comparability	Exposure			
NOS Case Control studies	Is the case definition adequate?	Representativeness of the cases	Selection of Controls	Definition of Controls	Comparability of cases and controls on the basis of the design or analysis	Ascertainment of exposure	Same method of ascertainment for cases and controls	Non-Response rate	Total 8/8
Song et al. [40]	☆	☆	0	☆	☆☆	NA	☆	0	6/8

Supplementary Table S2. Overview of the critical appraisal results. NA = not applicable.
