# Supplementary material for: Fistulizing Perianal Disease as a First Manifestation of Crohn’s Disease: A Systematic Review and Meta-Analysis
Source: J Clin Med. 2024 Aug 12;13(16):4734. doi: 10.3390/jcm13164734 (PMC11355404; doi:10.3390/jcm13164734)
Supplement: Supplementary file 1 [file jcm-13-04734-s001.zip › Supplementary Table S3 - (Converted) Time to diagnosis of CD after PAF manifestation.pdf]

Author	Median *	Median (m)	Mean	Mean (m)	Sample size
Göttgens et al. [33]	0.8 (0.2 - 2.7) y	9.6 (2.4 - 32.4)	14.8 (22.9) m	14.8 (22.9)	49
Mizushima et al. [36]	NR	NR	10.8 (15.8) m	10.8 (15.8)	188
Thia et al. [41]	38 (3 - 142) m	38 (3 - 142)	55 (39.96) m	55 (39.96)	15
Weng et al. [42]	NR	NR	1,611 (1,213) d	53.7 (40.4)	308
Ye et al. [44]	32 (2 - 361) m	32 (2 - 361)	106.75 (72.7) m	106.75 (72.7)	92

Supplementary Table S3. Time to diagnosis of CD after PAF manifestation as reported in studies and converted to a mean time to diagnosis in months using the method by Wan et al. [27]. * = median delay as was reported in the study; y = years; m = months; d = days; NR = not reported; sample size = number of patients in whom time to CD diagnosis was reported.
