# Supplementary figures and images for: Fistulizing Perianal Disease as a First Manifestation of Crohn’s Disease: A Systematic Review and Meta-Analysis
Source: J Clin Med. 2024 Aug 12;13(16):4734. doi: 10.3390/jcm13164734 (PMC11355404; doi:10.3390/jcm13164734)

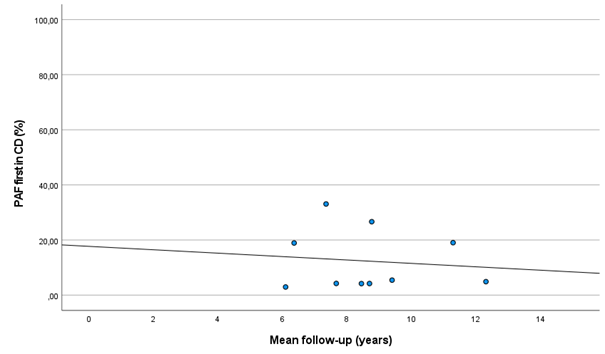

Supplement: Supplementary file 1 [file jcm-13-04734-s001.zip › Supplementary Figure S1 - Proportion of patients with a PAF prior to CD diagnosis plotted against mean follow-up.tif]
